# Supplementary material for: Modeling glioblastoma heterogeneity as a dynamic network of cell states
Source: Mol Syst Biol. 2021 Sep 16;17(9):e10105. doi: 10.15252/msb.202010105 (PMC8444284; doi:10.15252/msb.202010105)
Supplement: Supplementary file 5 — Source Data for Figure 3 [file MSB-17-e10105-s001.zip › Figure3A_sourcedata/GSEA_3065/hallmarks_state2.GseaPreranked.1623416269049/HALLMARK_ANGIOGENESIS.html]

Details for gene set HALLMARK\_ANGIOGENESIS[GSEA]

|  || Dataset | state2 |
| Phenotype | NoPhenotypeAvailable |
| Upregulated in class | na\_neg |
| GeneSet | HALLMARK\_ANGIOGENESIS |
| Enrichment Score (ES) | -0.84125215 |
| Normalized Enrichment Score (NES) | -2.374716 |
| Nominal p-value | 0.0 |
| FDR q-value | 0.0 |
| FWER p-Value | 0.0 |
Table: GSEA Results Summary

  

Fig 1: Enrichment plot: HALLMARK\_ANGIOGENESIS      
 Profile of the Running ES Score & Positions of GeneSet Members on the Rank Ordered List

  

| PROBE | GENE SYMBOL | GENE\_TITLE | RANK IN GENE LIST | RANK METRIC SCORE | RUNNING ES | CORE ENRICHMENT || 1 | CCND2 |  |  | 227 | 0.263 | 0.0343 | No |
| 2 | PDGFA |  |  | 1294 | 0.098 | -0.0570 | No |
| 3 | MSX1 |  |  | 2551 | 0.046 | -0.1801 | No |
| 4 | S100A4 |  |  | 4483 | 0.008 | -0.3834 | No |
| 5 | VEGFA |  |  | 4721 | 0.004 | -0.4078 | No |
| 6 | VAV2 |  |  | 4836 | 0.002 | -0.4194 | No |
| 7 | PTK2 |  |  | 5931 | -0.015 | -0.5321 | No |
| 8 | LPL |  |  | 8087 | -0.071 | -0.7452 | No |
| 9 | JAG1 |  |  | 8993 | -0.148 | -0.8085 | Yes |
| 10 | FGFR1 |  |  | 9034 | -0.155 | -0.7783 | Yes |
| 11 | LRPAP1 |  |  | 9144 | -0.176 | -0.7507 | Yes |
| 12 | FSTL1 |  |  | 9158 | -0.179 | -0.7123 | Yes |
| 13 | TNFRSF21 |  |  | 9223 | -0.202 | -0.6743 | Yes |
| 14 | COL5A2 |  |  | 9316 | -0.244 | -0.6299 | Yes |
| 15 | ITGAV |  |  | 9346 | -0.269 | -0.5732 | Yes |
| 16 | STC1 |  |  | 9378 | -0.314 | -0.5069 | Yes |
| 17 | TIMP1 |  |  | 9387 | -0.334 | -0.4335 | Yes |
| 18 | VCAN |  |  | 9396 | -0.364 | -0.3536 | Yes |
| 19 | APP |  |  | 9412 | -0.426 | -0.2606 | Yes |
| 20 | SPP1 |  |  | 9415 | -0.436 | -0.1641 | Yes |
| 21 | POSTN |  |  | 9437 | -0.750 | 0.0003 | Yes |
Table: GSEA details [plain text format]

  

Fig 2: HALLMARK\_ANGIOGENESIS: Random ES distribution      
 Gene set null distribution of ES for **HALLMARK\_ANGIOGENESIS**
